# Missing Person Protocol: Rapid Risk Assessment Re-Audit 2021

**DOI:** 10.1192/bjo.2022.294

**Published:** 2022-06-20

**Authors:** Juliette Fowler

**Affiliations:** NHS Grampian, Aberdeen, United Kingdom

## Abstract

**Aims:**

The Rapid Risk Assessment (RRA) has been a part of the Missing Person's Protocol since 2017 following a ward level intervention to try and provide as much information in as succinct a way as possible to the Police when a patient goes missing from the ward. This tool allows for rapid evaluation of a person's risk level on admission to hospital allowing consistent decisions to be made around risk to self and others, including physical risk and states why the risk level has been so set. In line with the National Framework for Missing Persons, a Return to Ward Interview is undertaken when a patient returns to the ward. The document is reviewed on a weekly basis at MDTs. The aim is to re-audit the extent to which the RRA within all wards at Royal Cornhill Hospital has been completed within the patients’ notes.

**Methods:**

- All General Adult (GAP), Older Adult (OAP) and Learning Disability Wards were audited for the level of completion of the RRA proforma.
- 10 sets of notes were audited in each ward (where possible).
- Data were gathered on a proforma for consistency looking at each area of the RRA: Patient Details, Brief Admission Details, Risk Level, Police Contact.

**Results:**

58 sets of patient notes were checked. 100% of notes contained the RRA proforma.

The average completion of all sections was 87.5%.

There has been a 21% improvement in completion of the RRA since the first audit in 2017. There was variability across the wards, but there has been a 14.5% improvement in completion of sections compared to the previous audit.

The Patient Details section of the RRA was the most fully completed area, The Brief Admission Details section was poorly completed and it is important to be able to give this information to the Police when they are contacted about a missing person.

**Conclusion:**

Across the wards, the data were less well completed by General Adult Psychiatry and best within Learning Disabilities. This is perhaps because of the higher turnover of patients but it would be interesting to consider the reasons for the disparity in data.

Improvement seems to have been driven by the teaching around the RRA and weekly review of the RRA at MDT

None of the wards audited had completed the Return to Ward Questionnaire. The ward staff made comment that the questions within this document are asked but more informally.

